# A detailed investigation of the porcine skin and nose microbiome using universal and *Staphylococcus* specific primers

**DOI:** 10.1038/s41598-018-30689-y

**Published:** 2018-08-24

**Authors:** Mikael Lenz Strube, Julie Elvekjær Hansen, Sophia Rasmussen, Karl Pedersen

**Affiliations:** National Veterinary Institute, Technical University of Denmark, Kemitorvet, building 202, DK-2800 Kgs Lyngby, Denmark

## Abstract

MRSA is an increasing problem in humans as well as livestock. The bacterial co-colonization of the skin in MRSA carriers has been poorly investigated and moreover, there have been no methods for high resolution investigations of the *Staphylococcus* genus apart from tediously culturing or doing multiple PCRs. On 120 samples from pig ear, skin and nose, we generated amplicons from the V1-V2 region of the 16S rRNA gene to gather an overview of the genus-level microbiome, along with using MRSA specific plates to count MRSA. In parallel with this, amplicons of the *tuf* gene were generated, targeting only a region of the *tuf* gene found only in the *Staphylococcus* genus. Using these methods, we determined a core microbiota across the healthy pig and determined the *Staphylococcus* genus to be dominated by *S. equorum*. Moreover, we found *Streptococcus* to be inversely associated with *Staphylococcus* and MRSA, suggesting a role for this genus in combating MRSA. In this work, we have thoroughly investigated the skin and nose microbiome of the pig and developed a high throughput method for profiling the *Staphylococcus* genus which we believe will be useful for further investigations.

## Introduction

During the last decade, a new strain of livestock-associated methicillin-resistant *Staphylococcus aureus* (LA-MRSA) has emerged worldwide in many different animal species e.g. cattle, poultry, mink, horses and especially pigs^1–3^. This strain, LA-MRSA CC398, is now widespread in Europe as well as in the Danish pig production^4,5^. At this point in time, the main issue of having LA-MRSA in production animals is the zoonotic risk it constitutes to farm workers^6^, although a spill-over to the general population may also occur^4^. It is unlikely that a complete elimination of LA-MRSA from the farm environment is possible without culling herds^4^, but an achievable ambition is perhaps to lower the level of LA-MRSA in farms. Previous studies have assessed the potential of possible interventions strategies, such implementation of disinfection and hygiene control measures^7^ or reduced usage of antimicrobials^8^, with inconclusive results. Radical measures have been implemented in the Norwegian pig production, which included a “search-and-destroy” policy, with depopulation and restocking of MRSA-free pigs. This method has shown to be effective in Norway, a country with low prevalence of MRSA in their pig production^9^, however impossible to implement in countries with high prevalence of LA-MRSA in pig farming such as Denmark^10^.

An alternative approach as intervention against LA-MRSA could be manipulation of the natural microbiome. It is known that the microbiome plays an important role in the health and disease of the host, and probiotics can in, the right amounts, confer considerable benefits to the host^11,12^. A study by^13^ found the human nasal microbiota not to be fixed by host genetics, and susceptible to environmental modifications. As concluded in the study, this perhaps allows for probiotics to be used in elimination of *S. aureus* nasal colonization or that a certain dermobiome selects for MRSA whereas another does not. A few animal studies have investigated the nasal microbiome in pigs. One study found a promising 20 bacterial candidates associated with non-carriage of *S. aureus* in the porcine nasal microbiome, including species from the family of *Leuconostocaceae* and *Lachnospiraceae*^14^. Another study found no significant difference in the microbiota of MRSA positive and negative pigs. However, they saw increased operational taxonomic units (OTUs) belonging to *Firmicutes* as main indicator of MRSA non-carriage, including *Staphylococcus* among others^15^. The approach used by Weese *et al*.^15^ did not allow for in depth resolution of the different staphylococcal species which, as pointed out by the authors, could be necessary to gain information regarding the potential protective effect against MRSA.

The use of the 16S rRNA gene is routinely used for profiling microbiotas due to its ubiquity and discriminatory power, but other genes, such as the Elongation Factor Thermo unstable (EF-Tu) gene (*tuf)*, may be useful for more targeted investigations. This gene universally conserved in bacteria, and codes for a protein that binds tRNA in the cytoplasm and mediates entry into the ribosome^16^. It has previously been used to distinguish *Lactobacillus* and *Bifidobacterium*^17^ as well as *Staphylococcus*^18,19^, but not yet in a high-throughput context.

In this study, we aimed to investigate the staphylococcal community in high resolution to identify which species dominate the pig nasal and skin microbiota and if any are associated with MRSA. As part of the instigation we have developed a high-throughput method based on selective primers targeting the *tuf* gene, e.g. an extension of the *tuf*-based classification used previously^18,19^. This enables one to achieve a large number of reads within the *Staphyloccocus*-genus, i.e. a large quantum of information, even at very low abundance. Swabs from clinical mink samples were included in the study as verification of the versatility of our approach. For cross-host verification, we included samples from both mink and pigs, as the major staphylococcal pathogens in mink are separate from the ones in pigs.

## Results

### The core microbiota of the skin, nose and ear

**Figure 1:**
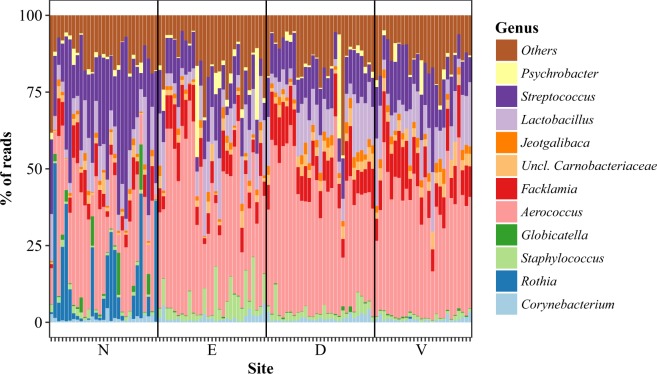
Barplot of amplicons from 16S rRNA gene sequencing. Each column corresponds to a single sample in which the relative abundance of bacterial genera is shown through color coding. N: nose, E: ear, D: dorsal skin surface, V: ventral skin surface.

The overall microbiota was assayed by sequencing of the V1-V2-amplicons, which after the entire bioinformatics pipeline, contained 32,857 classified reads per sample on average. The samples were fairly similar across sites and were dominated by the genera *Aerococcus* (36.2%), *Streptococcus* (15.9%), *Lactobacillus* (10.4%), *Facklamia* (8.7%), *Rothia* (3.2%) and *Staphylococcus* (2.7%) forming a core microbiota (see Fig. 1).

**Figure 2:**
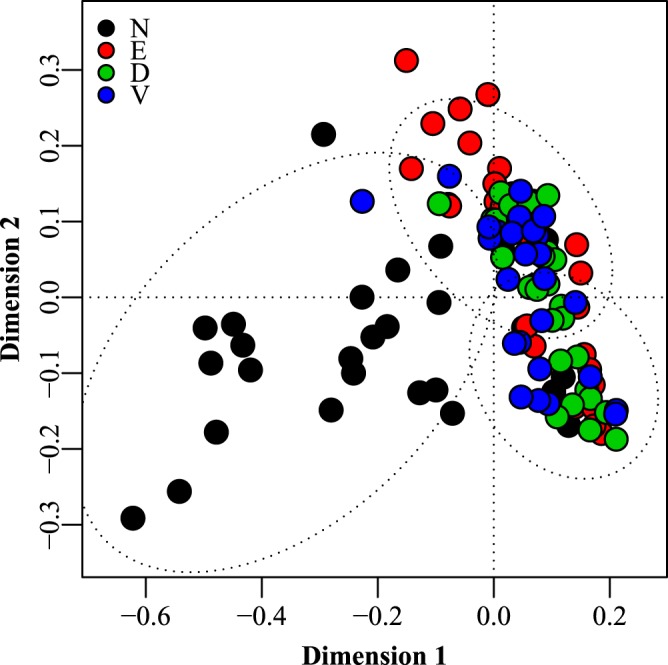
Principal coordinates analysis of amplicons from 16S rRNA gene sequencing. The multivariate ordination is based on the dissimilarity matrix derived from the relative abundance of bacterial genera across the samples. N: nose, E: ear, D: dorsal skin surface, V: ventral skin surface.

There were significant differences between sites according to multivariate analysis (P < 0.001 for ANOSIM, Adonis and PERMANOVA), as indicated in Figs 1 and 2. Pairwise comparisons using PERMANOVA showed that the ventral and dorsal surfaces were not significantly different, whereas the nose and the ear was significantly different from all other sites, the nose being the most dissimilar site (Figs 1 and 2). Canonical analysis of principal coordinates revealed that the genera separating the nose from the other sites were higher levels of *Streptococcus, Rothia* and *Globicatella* and lower levels of *Aerococcus* and *Facklamia*, whereas the ear was separated by higher levels of *Staphylococcus* and lower levels of *Facklamia* and *Streptococcus*.

**Figure 3:**
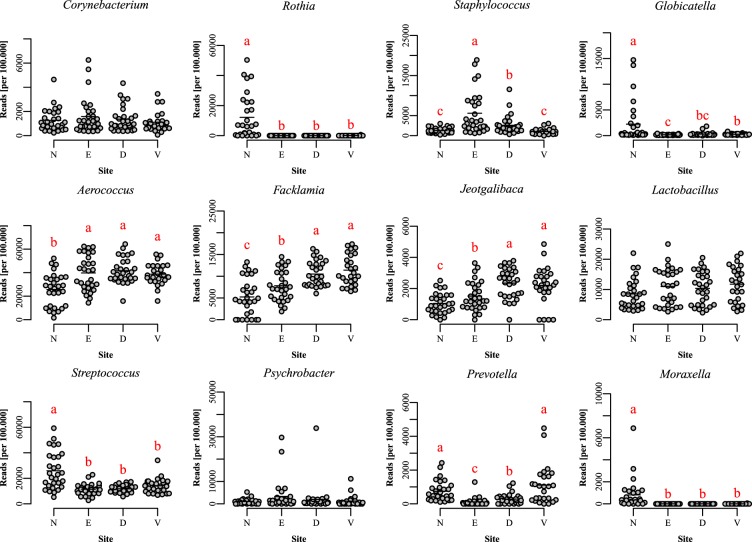
The most abundant and otherwise interesting bacterial genera from 16S rRNA gene sequencing. N: nose, E: ear, D: dorsal skin surface, V: ventral skin surface. Differences are tested with Kruskal-Wallis and, if significant, different medians are denoted by different letters. P-values are adjusted for multiple comparisons.

In univariate analysis, the nose uniquely harbored *Rothia* and had higher levels of *Streptococcus* and *Moraxella*, while levels of *Aerococcus*, *Facklamia* and *Jeotgalibaca* were lower (Fig. 3). The ears had the highest levels of *Staphylococcus*, but correspondingly lower levels of *Streptococcus* and *Prevotella*. The dorsal and ventral skin surface had higher levels of bacteria normally associated with the gut, such as *Prevotella*, *Bacteroides* and *Enterococcus*, although the dorsal surface was higher in *Staphylococcus*. The species within the *Staphylococcus* genus mapped almost exclusively to *S. equorum* or were unclassifiable beyond the genus level.

The Shannon index, a measure of diversity, was not different across groups (P = 0.95). The S*treptococcus* genus was inversely correlated with *Staphylococcus* (pearsons ρ = −0.38, P < 0.001).

### The *Staphylococcus* genus profiled by the *tuf*-gene

To gain a higher understanding of the species composition of the *Staphylococcus* genus in the samples, amplicons were generated from the *tuf* gene. The amplicons consisted exclusively of DNA classifiable to the *Staphylococcus* genus and primers were capable of amplifying the DNA of all tested species in the positive control, albeit with very different effectivity, e.g. *S. equorum* was positively biased and especially *S. sciuri, S. haemolyticus* and *S. epidermidis* was very negatively biased (See Supplementary Table S1). Using 16 S rRNA gene amplicons on the positive control did not find *S. delphini*, *S. xylosus* or *S. sciuri* and incorrectly found *S. saprophyticus*. 16S rRNA gene amplicons did however estimate more realistic proportions of the species herein, but when the *Staphylococcus* genus is a minority constituent, the species differentiation is low or non-existent.

**Figure 4:**
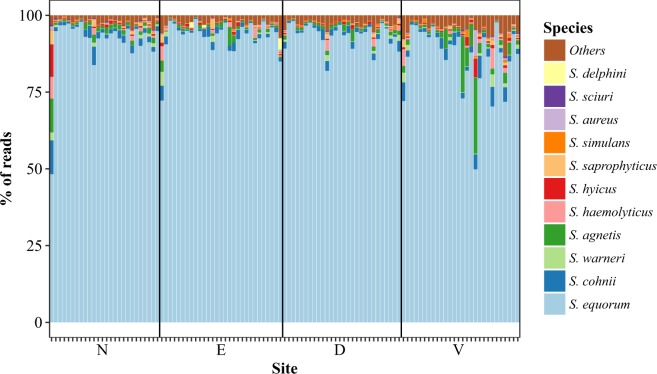
Composition of pig samples using *tuf* gene primers. Each column corresponds to a single sample in which the relative abundance of bacterial genera is shown through color coding. N: nose, E: ear, D: dorsal skin surface, V: ventral skin surface.

**Figure 5:**
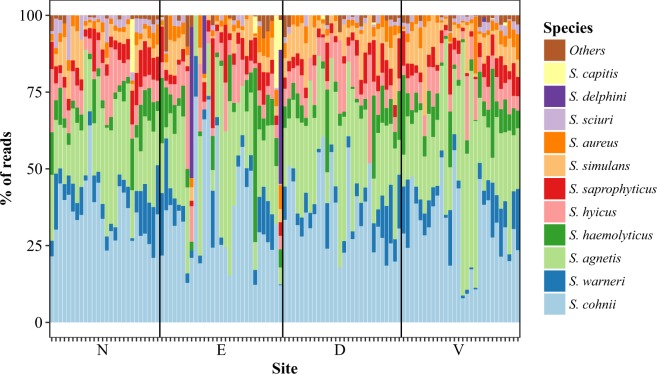
Composition of pig samples using *tuf* gene primers with *S. equorum* subtracted. Each column corresponds to a single sample in which the relative abundance of bacterial genera is shown through color coding. N: nose, E: ear, D: dorsal skin surface, V: ventral skin surface.

**Figure 6:**
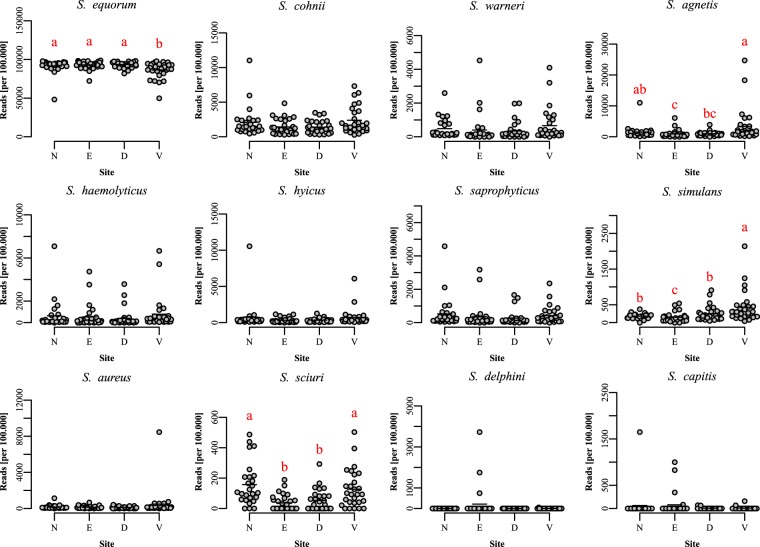
The most abundant and otherwise interesting staphylococcal species from *tuf* sequencing. N: nose, E: ear, D: dorsal skin surface, V: ventral skin surface. Differences are tested with Kruskal-Wallis and, if significant, different medians are denoted by different letters. P-values are adjusted for multiple comparisons.

In the animal samples, 31,508 reads were available for classification per sample on average, and 95–99% of these reads were classifiable to an unambiguous single species using blastn. Several *Staphylococcus* species were found in all samples (Figs 4 and 5), but all were heavily dominated by *S. equorum* followed by varying proportions of most species included in the positive control. *S. aureus* was detected in all samples. Overall, the ear was the most dissimilar site (Fig. 6), as *S. schleiferi*, *S. microti, S. simulans, S. hominis, S. lentus, S. sciuri* and *S. succinus* were relatively lowered here. Moreover, the ventral skin surface was lower in *S. equorum*, and correspondingly higher in *S. agnetis, S. simulans* and *S. sciuri*. Using a qPCR targeting the *S. equorum* SodA gene, the presence of *S. equorum* was confirmed in all samples. There was a linear correlation between qPCR estimates of *S. equorum* (pearsons ρ = 0.35, P < 0.001), although several ear samples were poorly described by this relation. Omission of ear samples showed a substantially higher correlation (pearsons ρ = 0.75, P < 0.001).

### MRSA levels on pig and mink

**Figure 7:**
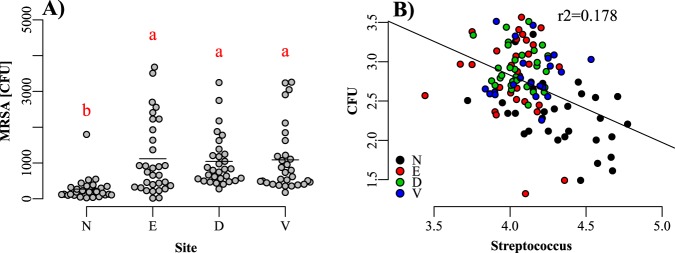
(**A**) MRSA counts by plating and the association to *Streptococcus* by V1-V2 sequencing. (**B**) Correlations of *Streptococcus* and MRSA CFU. N: nose, E: ear, D: dorsal skin surface, V: ventral skin surface. Differences are tested with Kruskal-Wallis and, if significant, different medians are denoted by different letters.

The presence of MRSA was assayed using MRSA selective plates. MRSA was present in all samples with levels around 10^3^ cfu/sample in ear and skin swab samples, whereas slightly lower levels, generally between 10^2^ and 10^3^ cfu/sample were recorded in nasal swab samples (Fig. 7A). MRSA plate counts was negatively correlated with *Streptococcus* (pearsons ρ = −0.42, P < 0.001) (Fig. 7B), but poorly related to total *Staphylococcus*, possibly reflecting that most of the staphylococci are *S. equorom*. We did not find any large correlations (pearsons ρ > 0.2) between the bacteria found using the *tuf-*primers and MRSA counts.

For mink, included for cross-validation of the used methods, the V1-V2 primers showed a diverse group of bacterial families, including *Staphylococcus*. The staphylococci in these V1-V2 samples were not classifiable at a species level or classified mainly as *S. pseudintermedius*. The *tuf* primers generated amplicons in most of the samples, mainly classified as *S. delphini* which is in agreement with classification by MALDI-TOF (data not shown). *S. delphini* was not detected using V1-V2 primers.

## Discussion

In this set of data, the microbiota of the nose, the outer ear and two sites of the skin were investigated using three different methods in order to investigate associations between the microbiota, MRSA and individual species of the *Staphylococcus* genus.

The overall skin and nasal microbiota was rich in *Aerococcus*, *Staphylococcus, Lactobacillus, Streptococcus, Facklamia* and *Rothia* (nose only), which is in some contrast to previous studies where bacteria belonging to the phylum *Proteobacteria* have dominated. One paper found a high abundance of the phylum *Proteobacteria* (including *Moraxella*, *Psychrobacter* and *Pseudomonas*) in the nose, whereas the present results are much higher in *Firmicutes*. The authors also found negative associations with MRSA for *Lactobacillus* and *Staphylococcus*, and proposed a more thorough investigation using specific primers^15^. A study looking at the tonsil microbiota of pigs also found high levels of *Proteobacteria*, especially *Pasteurella*, and an overall composition substantially different than in the present data, possibly explained by the different body site investigated^20^. A more recent paper reported high nasal levels of *Gammaproteobacteria*, mainly mapped to the genus *Moraxella*, which we also found abundantly in the nose. Compared to the present data, the paper also found similar levels of *Lactobacillus* and *Staphylococcus*^21^*. Moraxella* is well known as an inhabitant of mucosal surfaces^22^, and consequently, its dominance in nasal samples is not unexpected. Swe *et al*.^23^ investigated the microbiota of the ear thoroughly and found high levels of *Streptococcus, Lactobacillus* and *Corynebacterium*, but less *Aerococcus* and in contrast to our results, *Rothia* was found in the ear. *Rothia nasimurium* is a gram positive coccoid bacterium that was first described from the nose of healthy mice^24^, but it is found frequently in nasal swab samples from healthy pigs in our lab. It grows on MRSA2 agar with colonies similar to MRSA and thus constitutes a differential diagnostic issue. Regarding the dorsal and ventral skin of the animal, this has been investigated recently in a study by McIntyre *et al*.^25^, where high levels of *Firmicutes*, including *Ruminocococeae, Lachnospiraceae, Streptococcus* and *Prevotellaceae* were found and similar compositions were seen in the ventral and dorsal sites. Espinosa-Gongora *et al*.^14^ specifically investigated the nasal microbiota of MRSA carrier pigs vs. non-carriers, and apart from a core microbiota dominated by *Proteobacteria* and *Firmicutes*, species within *Lachnospiraceae* and *Leuconostoc* were shown to be differentially abundant in these animals. A differential analysis of carriers vs. non-carriers could not be carried out in the present data, as all animals were positive for MRSA in agreement with estimates of MRSA prevalence in Danish pigs^10^. The inverse relation of *Streptococcus* to MRSA and *Staphylococcus* observed in our data has previously been reported in Bessesen *et al*.^26^ where a significant negative relation between *Streptococcus mitis* and MRSA was found in human samples through 16S rRNA gene sequencing and confirmed with *in vitro* inhibition assays. In future work, individual strains of *Streptococcus* from the samples of interest should be isolated and assayed for possible antagonistic effects towards MRSA.

MRSA specific plates were used to enumerate MRSA as there currently is no reliable molecular technique to quantify MRSA in mixed samples. The use of plates unfortunately is sensitive to the load of bacteria in the samples, which may explain why MRSA loads were lower in the nose.

We sought to further elucidate the composition of the staphylococcal genera by the use of primers specific for the *tuf* gene, an approach already considered for *Staphylococcus* by previous papers^18,19^. The primers used by Martineu *et al*.^18^ were chosen for a metataxonomic approach as their product length was better suited for the 2 × 250bp MiSeq platform. The primers were entirely specific for staphylococci and were successful in amplifying all species tested, albeit with differing specificity. In contrast to non-specific primers, such as for the 16 S rRNA gene, the *tuf* primers will selectively amplify and provide resolution in samples very low in staphylococci, even to the degree where these are undetectable by 16S rRNA gene primers. The use of *tuf* primers also allows detection *S. delphini* as in mink. The primers cannot, though, distinguish between MRSA or methicillin sensitive *S. aureus*, as these differentiated by the presence of a mecA cassette and not a variation in the *tuf* gene. Inspection of the *tuf* gene of the negatively biased species revealed various levels of mismatch in the primers, e.g. 2 out of 5 sequences classified as *S. haemolyticus* in the database generated from NCBI had a single mismatch in both the forward and reverse primer. On the other hand, all 5 sequences classified as *S. saphrolyticus* were perfectly matched by both primers, although these were biased against in the sequencing. The reasons for the positive bias of *S. equorum* are less clear, as the region of the primer binding site of other negatively biased species such as *S. cohnii* or unbiased species such as *S. aureus* were identical to those in *S. equorum*. Ideally, the primers would amplify all staphylococcal species equally, which will require further development. Alternative *tuf* primers or use of the *SodA* gene as in Blaiotta *et al*.^27^ should be investigated. Further degeneration of the primers could be beneficial to encompass all species, although it is doubtful if all bias can be completely removed. The issue of different sequences classified as the same species (e.g. as for *S. haemolyticus*) further complicates intelligent primers design.

In the pigs, we observed very high levels of *S. equorum*, a species first found on the skin of healthy horses^28^ and described in the nose of pigs^29^, followed by *S. schleiferi, S. cohnii* and *S. microti*, as well as *S. aureus* in all samples, whereas the mink samples were dominated by *S. delphini*. The high prevalence of *S. equorum*, a species hitherto not having received much attention, was further investigated with a specific qPCR, showing that all samples were positive for *S. equorum* and that the relative abundance estimated from sequencing was in agreement with the relative abundance estimated by qPCR.

In conclusion, we have conducted a thorough investigation of the skin, nose and ear microbiota of the pig and implemented a high resolution method for elucidation of the *Staphylococcus* genus. The use of *tuf*-specific primers, despite not being entirely quantitative, allows for detection of individual *Staphylococcus*-species even in very low abundance, making future investigation of staphycoccal populations more straightforward.

We found that the microbiota of the nose differed significantly from the microbiota at the skin sites. The microbiome in all sites was dominated by *Aerococcus*, *Streptococcus*, *Lactobacillus*, *Facklamia*, *Rothia* and *Staphylococcus*, whereas the nose was enriched with *Streptococcus* and *Moraxella* and uniquely harboring *Rothia*. The staphylococcal population was heavily dominated by *S. equorum* followed by *S. schleiferi, S. cohnii* and *S. microti*, as well as *S. aureus*. In the nose, the level of MRSA was negatively correlated with the level of *Streptococcus* which makes *Streptococcus* a possible target of interest regarding manipulation of the natural microbiota towards an anti-MRSA environment.

## Materials and Methods

### Sample collection

Pig samples were obtained at a commercial slaughterhouse (Danish Crown, Ringsted, Denmark), immediately following controlled atmosphere stunning by CO_2_ but prior to euthanasia. Each animal (n = 30) was sampled behind one ear, in the nose and on the ventral and dorsal skin surface by use of individual ESwabs (Copan Diagnostics Inc., Murrieta, CA, USA) for a total of 120 samples. The ear was sampled by three gliding motions behind the pinna, the nose by a rotary motion ~1 cm inside each of the nares and on the two skin surfaces by three consecutive ~3 cm strokes in the same location. All handling of animals was in accordance with regulations from the Danish Ministry of Justice.

Mink samples, some positive and some negative for MRSA were acquired from routine diagnostics of clinical mink sent to DTU-VET. Swab samples from throat were obtained as described by Hansen *et al*.^2^.

A S*taphylococcus* control was made by a mixing cultures of *S. aureus* (both resistant and sensitive to methicillin)*, S. cohnii, S. delphini, S. equorum, S. epidermidis, S. haemolyticus, S. hyicus, S. schleiferi, S. sciuri, S. succinus*, and *S. xylosus* equally, which subsequently was subjected to the same purification, PCR-amplification and sequencing procedure as the pig and mink samples.

### MRSA enumeration by culturing

Samples were processed within 2 hours of sampling and were not centrifuged or filtered due to concerns regarding flocculation of staphylococci and/or adhesion to skin cells and dust particles. For direct quantification of MRSA, 100 µL sample material, undiluted and in 10^−1^ dilution, was plated on MRSA-selective plates (Brilliance MRSA2 agar, Oxoid, Basingstoke, UK). Plates were incubated at 37 °C overnight and suspected MRSA colonies were counted after 18–24 h. One colony from each positive sample was confirmed as MRSA by MALDI-TOF and PCR detection of the *mecA* and *nuc* genes^30^.

### Microbiota analysis and preparations

DNA from each sample was purified with a Maxwell® LEV Blood DNA Purification Kit (Promega Corporation, Madison, WI, USA). First, the swab was vigorously shaken at high speed to loosen the cells from the swab. The whole sample was then transferred to a 2 ml Eppendorf tube and centrifuged for 15 min at 20.000 g. The supernatant was removed and the pellet was incubated for 60 min at 37 °C with 100 µl of lysozyme mixture (20 mM Tris-HCl (pH 8), 2 mM EDTA, 1.2% Triton X, 200 µg/ml lysostaphin and 25 mg/ml lysozyme). Subsequently, the samples were mixed with 350 µl lysis buffer, and one 5 mm stainless steel bead (Qiagen GmbH, Hilden, Germany) was added to the samples followed by shaking on a Qiagen TissueLyser II (Qiagen GmbH, Hilden, Germany) for 2 min at 20 Hz. Samples were then incubated for 1 h at 56 °C with 30 µl proteinase K, 20 mg/ml. The DNA was then extracted on a Maxwell®16 Research Instrument System (Promega Corporation, Wisconsin, USA) according to the manufacturer’s instructions. The concentration of the DNA was quantified on a NanoDrop ND-1000 Spectrophotometer (NanoDrop Technologies, Wilmington, DE, USA). Negative controls were included for purification to control background contamination.

The V1-V2 regions of 16 S rRNA gene was amplified by PCR and sequenced as described by Strube *et al*.^31^ using primers V1V2Fw: 5′-AGA GTT TGA TCC TGG CTC AG-3′ and V1V2Rv: 5′-CTG CTG CCT YCC GTA-3′ tagged with hexameric barcodes and PCR conditions including 94 °C for 6 min; 30 cycles of 94 °C for 45 s, 57 °C for 45 s, and 72 °C for 90 s; and 72 °C for 10 min. The *tuf* gene amplicons were prepared using primers adapted from Martineau *et al*.^18^, TufFw: 5′-GGC CGT GTT GAA CGT GGT CAA ATC A-3′ and TufRv: 5′-TIA CCA TTT CAG TAC CTT CTG GTA A-3′, but were tagged with unique hexameric barcodes to allow for multiplexing of samples. The PCR program included 94 °C for 5 min and 30 cycles of 94 °C for 30 s, 58 °C for 45 s, 72 °C for 90 s, and 72 °C for 10 min (final extension). The negative controls from the DNA purification, were included in both PCRs. Specificity of the primers were tested by PCR on pure culture of *Rothia nasimurium, Corynebacterium sp., Clostridium perfringens, Propionibacterium acnes, Streptococcus suis, Escherichia coli* and *Lactobacillus casei*, all of which were negative. The resulting PCR products for both primer sets, included negative controls, were then analyzed on an Agilent 2100 Bioanalyzer using an AgilentDNA1000 kit (Agilent Technologies, Waldbronn, Germany) and further pooled in equimolar ratios (50 ng per barcoded sample). The pooled DNA was then purified of primers and detergents using a Qiagen MinElute PCR purification kit (Qiagen GmbH, Hilden, Germany) according to the manufacturer’s instructions. Amplicons were submitted to The National High-Throughput DNA Sequencing Centre at the University of Copenhagen, Denmark, for sequencing on an Illumina MiSeq 250PE platform. The V1-V2 amplicons were then merged, quality filtered, chimera-checked and mapped against the RDP-II SSU database using the BION-meta software (Danish Genome Institute, Aarhus, Denmark). The *tuf* gene amplicons were merged and quality filtered using BION-meta, clustered at 97% similarity with USEARCH^32^, *de-novo* chimera checked with UCHIME^33^ and taxonomically assigned using the blastn algorithm from command line BLAST with a custom made *tuf* database, using a word length of 22 and a minimum similarity of 90%. An alternative workflow using only USEARCH was also investigated, but was abandoned due to lower sensitivity. The *tuf* database was built by downloading all *Staphylococcus* genomes being either classified as “Complete genome” or “Scaffold” from NCBI Genbank (as indexed in ftp://ftp.ncbi.nlm.nih.gov/genomes/genbank/bacteria/assembly_summary.txt) followed by extraction of all CDS annotated as *tuf* genes using an in-house Perl- and bash-based script (available upon request). The *tuf* genes were then checked for correct length (between 1150 bp and 1200 bp) and used as a database for blastn. The *tuf* gene from *S. delphini* was added manually.

An *S. equorum* specific qPCR targeting the S. equorum sodA gene was carried out as described in Blaiotta *et al*.^27^ with the addition of QuantiTect SYBR Green (Qiagen, Hilden, Germany) using 25 ng DNA for each reaction. A standard curve of serial dilutions of *S. equorum* DNA was used to ensure amplification efficiency. The estimates of *S. equorum* from the qPCR, expressed in *S. equorum* DNA per 25 ng total DNA, was compared to the estimates of total S*taphylococcus* from 16S rRNA sequencing to further investigate the levels of *S. equorum*.

### Statistical analysis

All samples were normalized to 100,000 reads before further analysis and data is presented in its entirety when plotted. To avoid issues of normality, individual OTUs were compared using Kruskal-Wallis followed by Conover-Iman test if significant as implemented in the agricolae package^34^. The Shannon-index was used to estimate diversity and was tested with ANOVA. Multivariate patterns were visualized by principal coordinates analysis (PCoA) and tested for groupwise differences with ANOSIM, Adonis and PERMANOVA from the vegan package^35^, all using Bray-Curtis distances. Canonical analysis of principal coordinates was used to evaluate bacteria of interest. Correlations are calculated and plotted as log10 using Pearson correlations. P-values below 0.05 were considered significant, and were adjusted for multiple comparisons using Sidak-corrections^36^ when doing multiple univariate analyses. All tests were two-tailed.

### Ethics Approval and Consent To Participate

All handling of animals was in accordance with regulations from the Danish Ministry of Justice.

## Electronic supplementary material

Supplementary Table

Supplementary Dataset 1

## Data Availability

All sequence files, de-multiplexed, merged and quality-filtered, are available in the NCBI Sequence Read Archive (SRA) as part of the Bioproject found at https://www.ncbi.nlm.nih.gov/bioproject/PRJNA399517. Metadata is given in supplementary file 1.

## References

[1] Petinaki E, Spiliopoulou I (2012). Methicillin-resistant *Staphylococcus aureus* among companion and food-chain animals: impact of human contacts. Clin Microbiol Infect..

[2] Hansen JE (2017). Livestock-associated methicillin-resistant *Staphylococcus aureus* is widespread in farmed mink (Neovison vison). Vet. Microbiol..

[3] Cuny C (2010). Emergence of methicillin-resistant *Staphylococcus aureus* (MRSA) in different animal species. Int. J. Med. Microbiol..

[4] Stone, K. Risk Assessment on Methicillin-Resistant *Staphylococcus aureus* (MRSA), with a focus on Livestock-Associated MRSA, in the UK Food Chain. *Food Standards Agency* (2017).

[5] DANMAP. Use of antimicrobial agents and occurrence of antimicrobial resistance in bacteria from food animals, food and humans in Denmark. ISSN 1600-2032 (2015).

[6] Köck R (2014). The impact of zoonotic MRSA colonization and infection in Germany. Berl. Munch. Tierarztl. Wochenschr..

[7] Espinosa-Gongora C, Panduro P, Saxmose S (2013). Effect of a disinfectant powder on methicillin-resistant *Staphylococcus aureus* in pigs, bedding and air samples under simulated farm conditions. Pig J..

[8] Dorado-García A, Graveland H, Bos MEH, Verstappen KM (2015). Van Cleef BAGL, Kluytmans JAJW, *et al*. Effects of reducing antimicrobial use and applying a cleaning and disinfection program in veal calf farming: experiences from an intervention study to control livestock-associated MRSA. PLoS One..

[9] Grøntvedt CA (2016). Methicillin-resistant *Staphylococcus aureus* CC398 in humans and pigs in Norway: a “One Health” perspective on introduction and transmission. Clin. Infect. Dis..

[10] Ministry of Environment and Food of Denmark. MRSA Risiko og håndtering Rapport ved MRSA-ekspertgruppen. http://mfvm.dk/fileadmin/user_upload/MFVM/MRSA_rapport.pdf (2017).

[11] Ouwehand AC, Salminen S, Isolauri E (2002). Probiotics: an overview of beneficial effects. Antonie Van Leeuwenhoek..

[12] Cho JH, Zhao PY, Kim IH (2011). Probiotics as a dietary additive for pigs: A review. J. Anim. Vet. Adv..

[13] Liu CM (2015). *Staphylococcus aureus* and the ecology of the nasal microbiome. Sci. Adv..

[14] Espinosa-Gongora C, Larsen N, Schønning K, Fredholm M, Guardabassi L (2016). Differential analysis of the nasal microbiome of pig carriers or non-carriers of *Staphylococcus aureus*. PLoS One..

[15] Weese JS, Slifierz M, Jalali M, Friendship R (2014). Evaluation of the nasal microbiota in slaughter-age pigs and the impact on nasal methicillin-resistant *Staphylococcus aureus* (MRSA) carriage. BMC Vet. Res..

[16] Lathe WC, Bork P (2001). Evolution of *tuf* genes: Ancient duplication, differential loss and gene conversion. FEBS Lett..

[17] Ventura M, Canchaya C, Klaenhammer TR, Zink R (2003). Analysis, characterization, and loci of the *tuf* genes in *Lactobacillus* and *Bifidobacterium* species and their direct application for species identification. Appl. Environ. Microbiol..

[18] Martineau F (2001). Development of a PCR assay for identification of staphylococci at genus and species levels. J. Clin. Microbiol..

[19] Hwang SM, Kim MS, Park KU, Song J, Kim EC (2011). *tuf* gene sequence analysis has greater discriminatory power than 16S rRNA sequence analysis in identification of clinical isolates of coagulase-negative staphylococci. J. Clin. Microbiol..

[20] Lowe BA (2012). Defining the “core microbiome” of the microbial communities in the tonsils of healthy pigs. BMC Microbiol..

[21] Slifierz, M. J., Friendship, R. M. & Weese, J. S. Longitudinal study of the early-life fecal and nasal microbiotas of the domestic pig. *BMC Microbiol*. **1**5(184) 1–12 8 (2015) 10.1186/s12866-015-0512-7 PMC4578254 26391877

[22] Quinn, P. J. *et al*. Veterinary Microbiology and Microbial Disease, 2^nd^ Edition. (John Wiley & Sons, 2011).

[23] Swe PM, Zakrzewski M, Kelly A, Krause L, Fischer K (2014). Scabies mites alter the skin microbiome and promote growth of opportunistic pathogens in a porcine model. PLoS Negl. Trop. Dis..

[24] Collins MD, Hutson RA, Båverud V, Falsen E (2000). Characterization of a *Rothia*-like organism from a mouse: Description of *Rothia nasimurium* sp. nov. and reclassification of *Stomatococcus mucilaginosus* as *Rothia mucilaginosa* comb. nov. Int. J. Syst. Evol. Microbiol..

[25] McIntyre MK, Peacock TJ, Akers KS, Burmeister DM (2016). Initial characterization of the pig skin bacteriome and its effect on *in vitro* models of wound healing. PLoS One..

[26] Bessesen MT (2015). MRSA colonization and the nasal microbiome in adults at high risk of colonization and infection. J. Infect..

[27] Blaiotta G, Ercolini D, Mauriello G, Salzano G, Villani F (2004). Rapid and reliable identification of Staphylococcus equorum by a species-specific PCR assay targeting the sodA gene. Syst Appl Microbiol.

[28] Schleifer KH, Kilpper-Bälz R, Devriese LA (1984). *Staphylococcus arlettae* sp. nov., *S. equorum* sp. nov. and *S. k1oosii* sp. nov.: Three new coagulase-negative, novobiocin-resistant species from animals. Syst. Appl. Microbiol..

[29] Tulinski P (2012). Methicillin-resistant coagulase-negative staphylococci on pig farms as a reservoir of heterogeneous staphylococcal cassette chromosome mec elements. Appl Environ Microbiol.

[30] Maes N, Magdalena J, Rottiers S, De Gheldre Y, Struelens MJ (2002). Evaluation of a triplex PCR assay to discriminate *Staphylococcus aureus* from coagulase-negative staphylococci and determine methicillin resistance from blood cultures. J. Clin. Microbiol..

[31] Strube ML, Ravn HC, Ingerslev H-C, Meyer AS, Boye M (2015). *In situ* prebiotics for weaning piglets: *in vitro* production and fermentation of potato galacto-rhamnogalacturonan. Appl. Environ. Microbiol..

[32] Edgar RC (2010). Search and clustering orders of magnitude faster than BLAST. Bioinformatics..

[33] Edgar RC, Haas BJ, Clemente JC, Quince C, Knight R (2011). UCHIME improves sensitivity and speed of chimera detection. Bioinformatics..

[34] de Mendiburu, F. agricolae: Statistical Procedures for Agricultural Research. Available from: https://cran.r-project.org/package=agricolae (2016).

[35] Oksanen, J. *et al*. vegan: Community Ecology Package. Available from: https://cran.r-project.org/package=vegan (2016).

[36] Šidák Z (1967). Rectangular confidence regions for the means of multivariate normal distributions. J. Am. Stat. Assoc..

