# Supplementary material for: A detailed investigation of the porcine skin and nose microbiome using universal and Staphylococcus specific primers
Source: Sci Rep. 2018 Aug 24;8:12751. doi: 10.1038/s41598-018-30689-y (PMC6109091; doi:10.1038/s41598-018-30689-y)
Supplement: Supplementary file 1 — Supplementary Table [file 41598_2018_30689_MOESM1_ESM.docx]

**A detailed investigation of the porcine skin and nose microbiome using universal and *Staphylococcus* specific primers**

Mikael Lenz Strube^a^*, Julie Elvekjær Hansen^a^, Sophia Rasmussen^a^ and Karl Pedersen^a^

^a^National Veterinary Institute, Technical University of Denmark, Kemitorvet, building 202, DK-2800 Kgs Lyngby, Denmark.

*Corresponding author: [milst@vet.dtu.dk](mailto:milst@vet.dtu.dk)

*Current address: DTU Bioengineering, Technical University of Denmark, Søltofts Plads, Building 221, DK-2800 Kgs. Lyngby, Denmark.

Mikael Lenz Strube: [milst@vet.dtu.dk](mailto:milst@vet.dtu.dk)

Julie Elvekjær Hansen: [juhan@vet.dtu.dk](mailto:juhan@vet.dtu.dk)

Sophia Rasmussen: [sopr@vet.dtu.dk](mailto:sopr@vet.dtu.dk)

Karl Pedersen: [kape@vet.dtu.dk](mailto:kape@vet.dtu.dk)

Supplementary Figures and Table

Table S1. The positive control included for 16S rRNA gene and *tuf* gene sequencing. Included are the expected and realized proportions (%) in each sample. Each column corresponds to one sample.

|  | **Expected** | **tufSTD** | **tufSTD** | **tufSTD** | **16sSTD** | **16sSTD** |
| --- | --- | --- | --- | --- | --- | --- |
| ***S. aureus*** | 16.7 | 19.5 | 18.7 | 19.3 | 15.8 | 15.1 |
| ***S. cohnii*** | 8.3 | 4.0 | 4.0 | 3.9 | 4.1 | 4.3 |
| ***S. delphini*** | 8.3 | 5.4 | 7.2 | 6.9 | 0.0 | 0.0 |
| ***S. epidermidis*** | 8.3 | 1.6 | 1.4 | 1.6 | 8.8 | 8.6 |
| ***S. equorum*** | 8.3 | 36.5 | 37.4 | 37.6 | 22.1 | 22.7 |
| ***S. haemolyticus*** | 8.3 | 0.8 | 0.8 | 1.0 | 6.1 | 5.9 |
| ***S. hyicus*** | 8.3 | 15.6 | 13.8 | 14.1 | 9.9 | 9.8 |
| ***S. pseudintermedius*** | 0.0 | 0.3 | 0.2 | 0.1 | 7.1 | 7.1 |
| ***S. saprophyticus*** | 0.0 | 0.2 | 0.3 | 0.1 | 4.3 | 4.3 |
| ***S. schleiferi*** | 8.3 | 10.1 | 10.4 | 8.6 | 9.8 | 9.8 |
| ***S. sciuri*** | 8.3 | 0.3 | 0.3 | 0.5 | 0.0 | 0.0 |
| ***S. succinus*** | 8.3 | 2.6 | 2.7 | 3.1 | 7.2 | 6.7 |
| ***S. xylosus*** | 8.3 | 2.8 | 2.6 | 2.8 | 0.0 | 0.0 |
| **Uncl. *Staphylococcus*** | 0.0 | 0.0 | 0.0 | 0.0 | 4.0 | 4.8 |
| **Others** | 0.0 | 0.4 | 0.2 | 0.3 | 0.8 | 1.0 |
